# Laceration of the Left Hepatic Vein Following Cardiopulmonary Resuscitation

**DOI:** 10.5334/jbsr.4054

**Published:** 2025-09-10

**Authors:** Femke Vrijdag, Joke Meersschaert

**Affiliations:** 1 AZ St. Jan Brugge, KU Leuven, Belgium; 2 AZ St. Jan Brugge, Belgium

**Keywords:** cardiopulmonary resuscitation, hemoperitoneum, hemorrhage, left hepatic vein

## Abstract

*Teaching point:* Laceration of the left hepatic vein is a rare but potentially fatal complication of cardiopulmonary resuscitation (CPR) and should be considered in patients with unexplained hemodynamic instability following resuscitation.

## Case History

**Figure 1:**
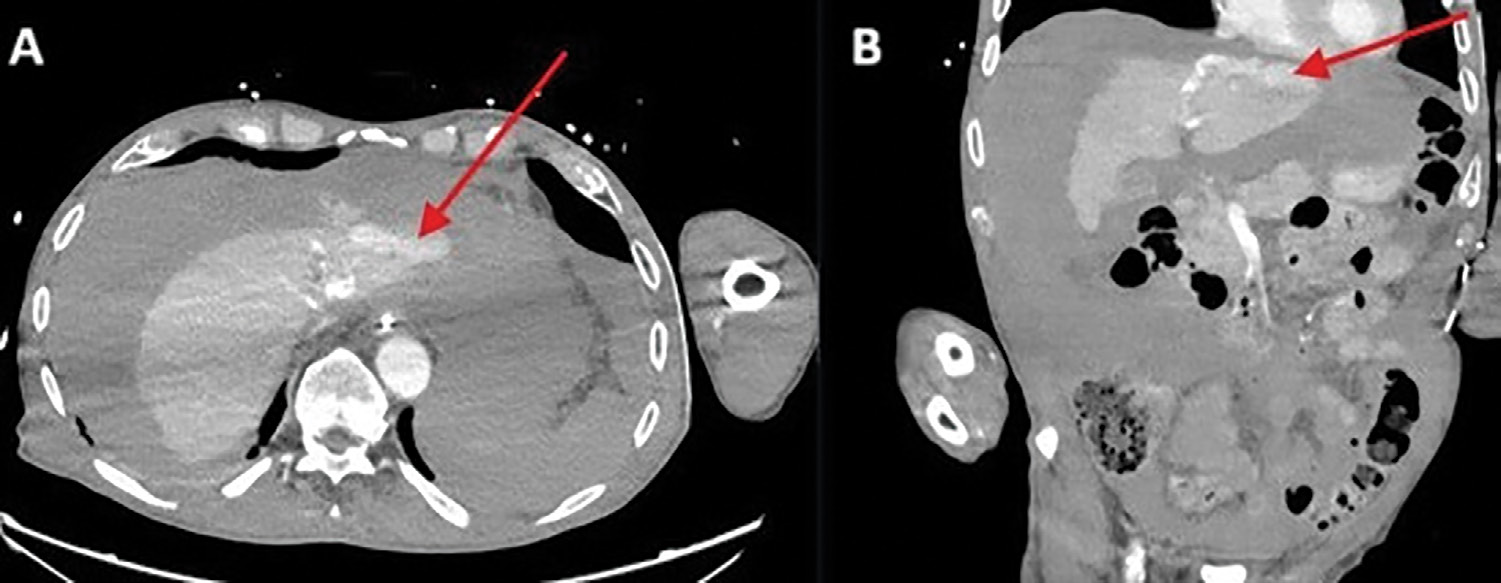
Active venous contrast extravasation against the left hepatic lobe.

A 78-year-old man was referred to the emergency department for cardiac arrest. Return of spontaneous circulation (ROSC) was achieved following manual cardiopulmonary resuscitation (CPR). Coronary angiography revealed three-vessel disease; the procedure was largely challenging due to a difficult femoral artery access by pronounced calcified atheromatosis. In the following hours, the patient developed progressive hemodynamic instability, accompanied by a decline in haemoglobin. An abdominal bed-side ultrasound revealed diffuse hyperechogenic fluid in the perihepatic, perisplenic, and lateral areas, as well as in the Douglas pouch, indicating diffuse hemoperitoneum. Computer tomography angiography (CTA) of the abdomen revealed active venous contrast extravasation against the left hepatic lobe (Figure 1 A, B; red arrow).

**Figure 2:**
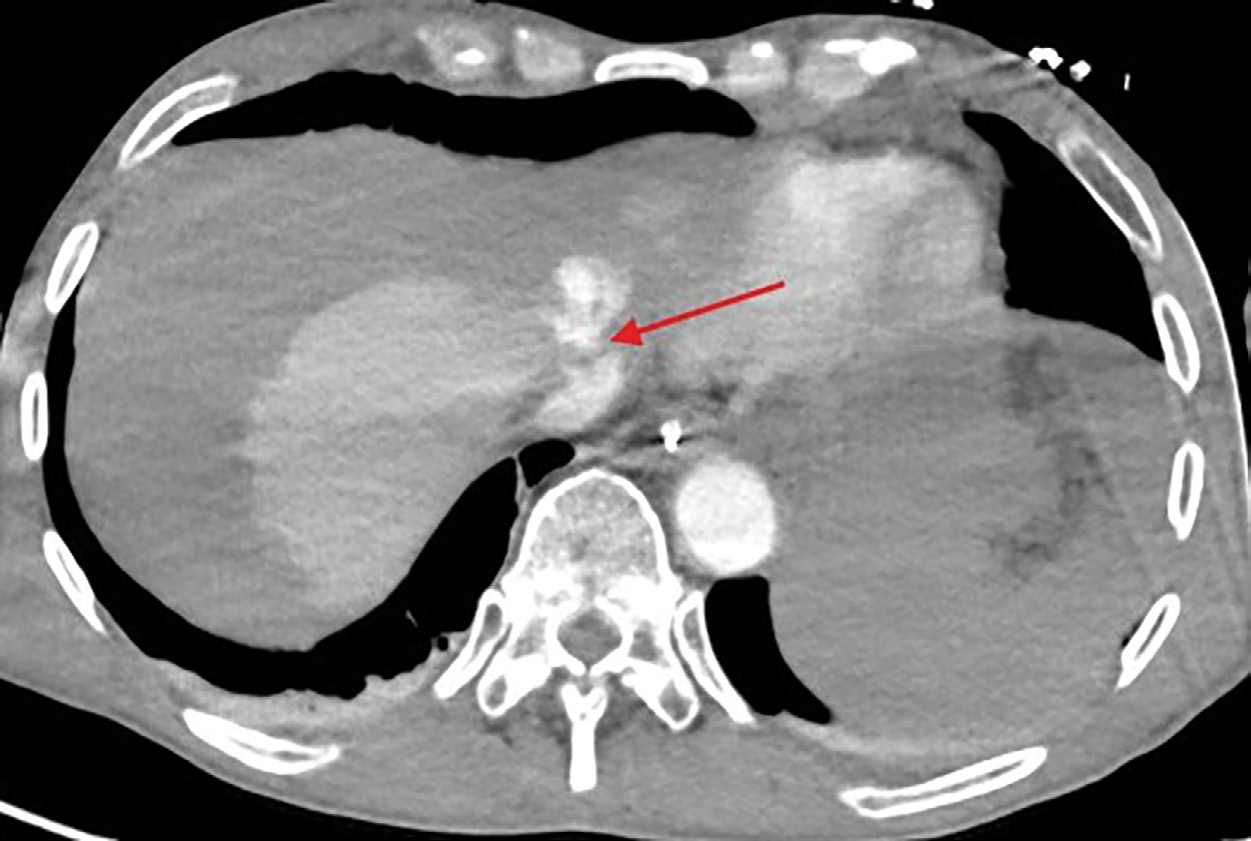
Lacerated left hepatic vein at the confluence with the vena cava.

The bleeding was most likely originating from the lacerated left hepatic vein, at the confluence with the vena cava (Figure 2, red arrow).

**Figure 3:**
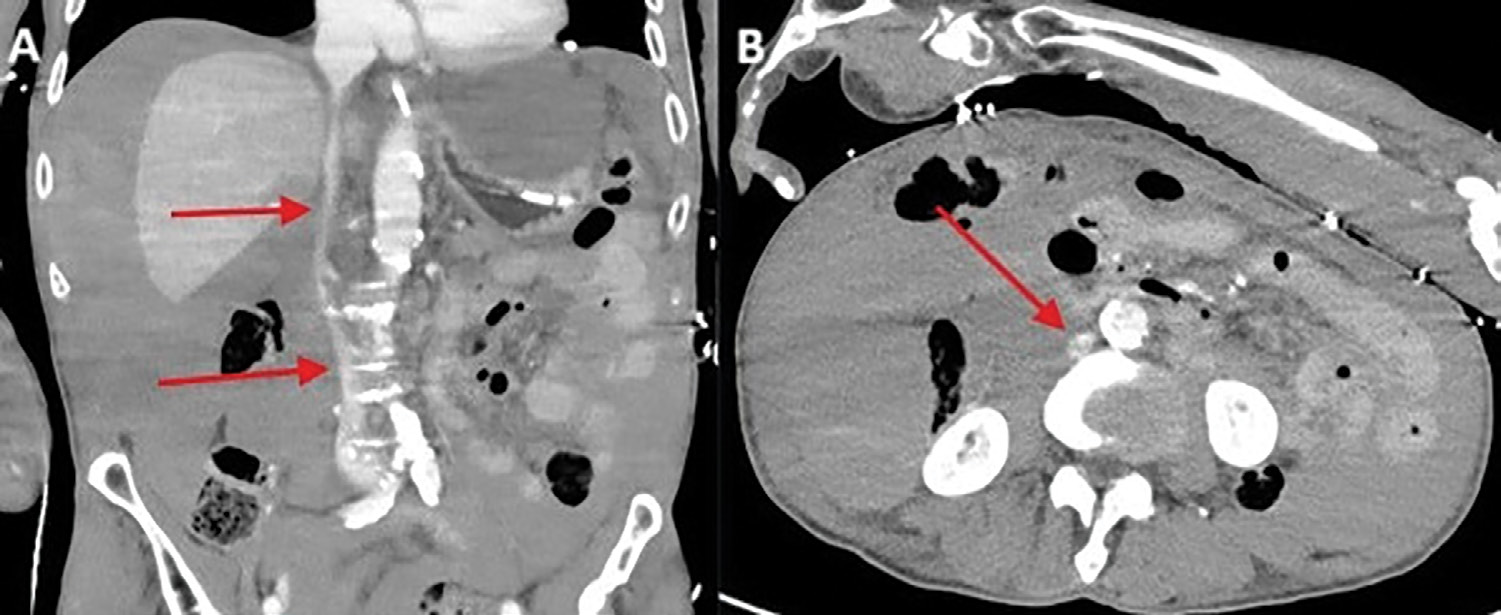
Gracile appearance of the inferior vena cava and the major pelvic veins.

A large amount of blood was present intraperitoneally. In addition, the inferior vena cava and the major pelvic veins appeared gracile consistent with hypovolemic shock (Figure 3 A, B; red arrow). The patient’s condition further worsened with a fatal outcome a few hours later.

## Comment

Chest compressions are a fundamental part of cardiopulmonary resuscitation (CPR), aimed at restoring circulation in cardiac arrest. Current guidelines recommend a compression depth of 5 cm at a rate of 100–120 per minute, with hand placement on the lower half of the sternum. While rib (28–85%) and sternal (30–71%) fractures are the most commonly reported complications, visceral injuries, though rare, can have significant clinical consequences [1]. Among these, liver lacerations are the most frequent, with an incidence of approximately 0.6% [1]. Such injuries are often underdiagnosed, particularly in the acute phase, as post-resuscitation patients are typically unresponsive and early signs of intra-abdominal bleeding may be subtle or delayed. Risk factors include improper hand placement, hepatomegaly due to liver and heart failure, and anticoagulation [1]. The left hepatic lobe is more frequently affected, likely due to its anatomical proximity to the sternum. Automated chest compression devices (e.g., CORPULS, LUCAS) improve the consistency and quality of compressions, however, they also carry a risk of visceral trauma [1]. Early recognition of liver injury is essential but challenging. Falling hematocrit levels may be a clinical indication, while bedside ultrasound offers a rapid, non-invasive method to assess free intra-abdominal fluid. Ultimately, contrast-enhanced triphasic CT remains the most effective modality for identifying and localizing active bleeding and vascular injury.
